# Direction- and distance-dependent interareal connectivity of pyramidal cell subpopulations in the rat frontal cortex

**DOI:** 10.3389/fncir.2013.00164

**Published:** 2013-10-11

**Authors:** Yoshifumi Ueta, Yasuharu Hirai, Takeshi Otsuka, Yasuo Kawaguchi

**Affiliations:** ^1^Division of Cerebral Circuitry, National Institute for Physiological SciencesOkazaki, Japan; ^2^Japan Science and Technology Agency, Core Research for Evolutional Science and TechnologyTokyo, Japan; ^3^Department of Physiological Sciences, Graduate University for Advanced Studies (SOKENDAI)Okazaki, Japan

**Keywords:** motor cortex, orbitofrontal cortex, posterior parietal cortex, perirhinal cortex, commissural, corticostriatal, corticopontine, corticothalamic

## Abstract

The frontal cortex plays an important role in the initiation and execution of movements via widespread projections to various cortical and subcortical areas. Layer 2/3 (L2/3) pyramidal cells in the frontal cortex send axons mainly to other ipsilateral/contralateral cortical areas. Subpopulations of layer 5 (L5) pyramidal cells that selectively project to the pontine nuclei or to the contralateral cortex [commissural (COM) cells] also target diverse and sometimes overlapping ipsilateral cortical areas. However, little is known about target area-dependent participation in ipsilateral corticocortical (iCC) connections by subclasses of L2/3 and L5 projection neurons. To better understand the functional hierarchy between cortical areas, we compared iCC connectivity between the secondary motor cortex (M2) and adjacent areas, such as the orbitofrontal and primary motor cortices, and distant non-frontal areas, such as the perirhinal and posterior parietal cortices. We particularly assessed the laminar distribution of iCC cells and fibers, and identified the subtypes of pyramidal cells participating in those projections. For connections between M2 and frontal areas, L2/3 and L5 cells in both areas contributed to reciprocal projections, which can be viewed as “bottom-up” or “top-down” on the basis of their differential targeting of cortical lamina. In connections between M2 and non-frontal areas, neurons participating in bottom-up and top-down projections were segregated into the different layers: bottom-up projections arose primarily from L2/3 cells, while top-down projections were dominated by L5 COM cells. These findings suggest that selective participation in iCC connections by pyramidal cell subtypes lead to directional connectivity between M2 and other cortical areas. Based on these findings, we propose a provisional unified framework of interareal hierarchy within the frontal cortex, and discuss the interaction of local circuits with long-range interareal connections.

## INTRODUCTION

Unlike cortical neurons in primary sensory areas, neurons in the frontal cortex can sustain persistent activity to encode specific information without external inputs, which may be supported by excitatory reverberation of (i) local recurrent connections among pyramidal cells; (ii) thalamocortical loops strongly influenced by the basal ganglia and cerebellum; and (iii) reciprocal interareal loops (Wang, 2001; Arnsten et al., 2012). Therefore, to understand the functional operation of the frontal cortex, it is crucial to reveal the formation rules for its corticocortical connections, as well as the relationships between pyramidal cells sending information to the thalamus, basal ganglia, and cerebellum, and those projecting to various cortical areas (Veinante and Deschênes, 2003).

The rat frontal cortex can be divided into three regions: the motor, orbitofrontal (OFC), and medial prefrontal cortices (Uylings et al., 2003; Gabbott et al., 2005; Hoover and Vertes, 2007). The motor cortex, which sends axons to the spinal cord, is further divided into the rostral secondary motor area (M2) and the caudal primary motor area (M1), which can be distinguished from M2 on the basis of lower stimulation thresholds for movement-evoking intracortical microstimulation and weaker immunolabeling for the neurofilament heavy chain (NF-H) (Brecht et al., 2004; Ueta et al., 2013). We have previously characterized two neuronal subtypes of M2 layer 5 (L5) pyramidal cells based on their long-distance axonal collateralizations to subcortical areas and their intracortical connectivity: corticopontine (CPn) cells that project to ipsilateral pontine nuclei and commissural (COM) cells that project to the contralateral cortex (Morishima and Kawaguchi, 2006; Otsuka and Kawaguchi, 2008, 2011; Morishima et al., 2011; Hirai et al., 2012; Ueta et al., 2013). Furthermore, we recently found that M2 projections preferentially innervate upper layer 1 (L1a), rather than lower L2/3 (layer 2/3) (L2/3b) of M1, whereas M1 efferents preferentially innervate L2/3b rather than L1a of M2 (Ueta et al., 2013). By analogy with the directionality of interareal connection demonstrated between visual cortices, this organization provides an anatomical basis for the “top-down” influence from M2 to M1 and the “bottom-up” influence from M1 to M2 (Coogan and Burkhalter, 1993; Dong et al., 2004).

Areas in the frontal cortex make reciprocal ipsilateral corticocortical (iCC) connections with multiple frontal cortical areas, as well as with distant non-frontal areas (Reep et al., 1990; Condé et al., 1995; Hoover and Vertes, 2007, 2011; Hira et al., 2013). In this study, we examine iCC organization in the fontal cortex and reveal the laminar distribution and subtype specificity of pyramidal cells involved in iCC connections between M2 and its target areas.

We demonstrate that the laminar pattern of iCC projections, and the relative participation of L5 CPn and COM cell subtypes among these projections, are specific to the pair of cortical areas involved as well as the direction of connectivity between the two areas. We outline a unified framework to understand the iCC connections of the frontal cortex with adjacent and distant areas that incorporates differences in top-down and bottom-up connectivity between areas.

## MATERIALS AND METHODS

### ANIMALS

Wistar rats (Charles River Laboratories Japan, Inc., Tsukuba, Japan) of either sex that were 19–23 days or 4–7 weeks old were used for physiological and histological experiments, respectively. Vesicular gamma-aminobutyric acid (GABA) transporter (VGAT)-Venus transgenic rats, which express the fluorescent protein Venus in GABAergic cells, were used to identify GABAergic cells (Uematsu et al., 2008). VGAT-Venus transgenic rats were generated by Drs. Y. Yanagawa, M. Hirabayashi, and Y. Kawaguchi at the National Institute for Physiological Sciences with pCS2-Venus that was provided by Dr. A. Miyawaki. VGAT-Venus rats are distributed by the National BioResource Project for the Rat in Japan^1^. All experiments were conducted in compliance with the guidelines of the Institutional Animal Care and Use Committee of the National Institutes of Natural Sciences.

### IMMUNOHISTOCHEMICAL IDENTIFICATION OF THE LAMINAR STRUCTURE IN FRONTAL CORTEX

Wistar rats were deeply anesthetized with sodium pentobarbital [60 mg/kg, intraperitoneal (i.p.)] and perfused transcardially with a prefixative [250 mM sucrose and 5 mM MgCl_2_ in 0.02 M phosphate-buffered (PB) saline, pH 7.4] followed by a fixative (4% paraformaldehyde and 0.2% picric acid in 0.1 M PB solution), and post-fixed within 30 min at room temperature. The brain was obliquely cut (Kawaguchi et al., 1989) into 20-μm sections using a vibratome (Leica Microsystems Inc., Buffalo Grove, IL, USA). Sections were incubated overnight at 4°C with a mouse monoclonal antibody against neuronal nuclei (NeuN; MAB377, EMD Millipore Corporation, Billerica, MA, USA; 1:5000) and a rabbit polyclonal antibody against calbindin D-28K (CB-38a, Swant, Marly, Switzerland; 1:2000) in 0.05 M Tris-buffered saline (TBS) containing 10% normal goat serum, 2% bovine serum albumin, and 0.5% Triton X-100. After washing in TBS, the sections were reacted with secondary antibodies conjugated to Alexa Fluor 488 (for NeuN; Life Technologies Corporation, Grand Island, NY, USA; 1:200) and Alexa Fluor 594 (for calbindin; Life Technologies Corporation; 1:200) for 2–3 h at room temperature. Adjacent sections were incubated overnight at 4°C with a guinea pig polyclonal antibody against vesicular glutamate transporter type 2 (VGluT2; AB2251, EMD Millipore Corporation; 1:5000) and a rat monoclonal antibody against chicken ovalbumin upstream promoter transcription factor-interacting protein 2 (Ctip2; ab18465, Abcam plc, Cambridge, UK; 1:500). After washing in TBS, the sections were reacted with secondary antibodies conjugated to Alexa Fluor 594 (for VGluT2) and Alexa Fluor 488 (for Ctip2). The sections were mounted on glass slides, coverslipped with Prolong gold antifade reagent (Life Technologies Corporation), and observed with epifluorescence.

### GABAergic CELL IDENTIFICATION AMONG NeuN-POSITIVE CELLS

VGAT-Venus transgenic rats were deeply anesthetized with sodium pentobarbital and perfused transcardially with a prefixative, which was followed by a fixative (4% paraformaldehyde, 0.1% glutaraldehyde, and 0.2% picric acid in 0.1 M PB solution). After post-fixation lasting 2 h at room temperature or overnight at 4°C, the brain was obliquely cut into 8-μm sections on a cryostat (Leica Microsystems Inc.). Sections were incubated overnight at 4°C with a mouse monoclonal antibody against NeuN (1:3000), a chicken polyclonal antibody against GFP/Venus (ab13970, Abcam plc; 1:1000), a guinea pig polyclonal antibody against VGluT2 (1:1500), and a rabbit polyclonal antibody against calbindin D-28K (1:2000) in 0.05 M TBS containing 10% normal goat serum, 2% bovine serum albumin, and 0.2% Triton X-100. After washing in TBS, sections were reacted with secondary antibodies conjugated to Alexa Fluor 594 (for NeuN), Alexa Fluor 488 (for both Venus and VGluT2), and Alexa Fluor 350 (for calbindin) for 2–3 h at room temperature. It was possible to discriminate the staining patterns between Venus (somata) and VGluT2 (fibers) at the same fluorescence.

### Ctip2-POSITIVE CELL IDENTIFICATION AMONG NON-GABAergic NEURONS IN L5

VGAT-Venus transgenic rats were deeply anesthetized with sodium pentobarbital and perfused with a prefixative, which was followed by a fixative (4% paraformaldehyde and 0.2% picric acid in 0.1 M PB solution). The brain was obliquely cut into 20-μm sections using a vibratome. Sections were incubated overnight at 4°C with a mouse monoclonal antibody against NeuN (1:1000), a chicken polyclonal antibody against GFP/Venus (1:1000), a guinea pig polyclonal antibody against VGluT2 (1:1500), and a rat monoclonal antibody against Ctip2 (1:500) in 0.05 M TBS containing 10% normal goat serum, 2% bovine serum albumin, and 0.2% Triton X-100. After washing in TBS, sections were reacted with secondary antibodies conjugated to biotin (for NeuN), Alexa Fluor 488 (for Venus), Alexa Fluor 488 (for VGluT2), and Alexa Fluor 594 (for Ctip2) for 2–3 h at room temperature. The NeuN signal was detected by further incubation with Alexa Fluor 350-conjugated streptavidin (Life Technologies Corporation; 1:200). Ctip2-positive and Ctip2-negative cells were counted among the Venus-negative and NeuN-positive cells separately in L5a and the upper and lower halves of L5b.

### RETROGRADE LABELING OF CPn CELL CLASSES

Animals were anesthetized with a mixture of ketamine (40 mg/kg, i.p.) and xylazine (4 mg/kg, i.p.) followed by an injection of glycerol (0.6 g/kg, i.p.) and dexamethasone (1 mg/kg, intramuscular) before being placed in a stereotaxic apparatus. Two tracers were used: Fast Blue (Dr. Illing GmbH and Co. KG, Groß-Umstadt, Hesse, Germany; 2% in distilled water) and Alexa Fluor 555-conjugated cholera toxin subunit B (CTB555; Life Technologies Corporation; 0.2% in distilled water). One or two fluorescent tracers with different excitations were injected into one or two target areas by pressure injection (PV820 Pneumatic PicoPump, World Precision Instruments, Inc., Sarasota, FL, USA) using glass pipettes (tip diameter, 50–100 μm; 100 nL in total).

Pontine nuclei were injected with Fast Blue (5.8–6 mm posterior to bregma, 0.8 mm lateral to the midline, 7.2–7.8 mm depth from the pial surface). The upper cervical cord was injected with Fast Blue or CTB555 (C1–2 segments). Ventral thalamic nuclei were injected with CTB555 (2–2.6 mm posterior to bregma, 1.4–2 mm lateral to the midline, and 5.4–5.8 mm from the surface). For superior colliculus injections, the cerebral cortex just above the superior colliculus was entirely removed by suction; CTB555 was applied vertically at two sites (6.5 mm posterior to bregma, 1.5 and 2 mm lateral to the midline, and 0.6, 0.8, and 1 mm from the surface of the superior colliculus). Because M2-derived anterogradely labeled fibers innervated intermediate and deep, but not superficial, zones of the superior colliculus (data not shown), the tracer was injected at a slightly deeper part of the superior colliculus. In each case, a total tracer volume of 100 nL was injected.

After a survival period of 4–6 days, the animals were deeply anesthetized with sodium pentobarbital and perfused transcardially with a prefixative, followed by a fixative (4% paraformaldehyde and 0.2% picric acid in 0.1 M PB solution). Using a vibratome, the frontal cortex was cut obliquely into 20-μm sections to observe labeled cells, and the brainstem was cut sagittally into 20- or 50-μm sections to confirm the injection sites. Every four serial cortical sections were collected as a set. In each set, the first or third section was used to count labeled cells. The others were used for the determination of cortical areas and layers.

### RETROGRADE LABELING OF iCC CELLS

CTB555 was injected into M2, OFC, and the posterior parietal cortex (PPC) by pressure injection (PV820) using glass pipettes (tip diameter, 50–100 μm; 100 nL in total). After a survival period of 4–6 days, the animals were deeply anesthetized with sodium pentobarbital and perfused with a prefixative followed by a fixative (4% paraformaldehyde and 0.2% picric acid in 0.1 M PB solution). After post-fixation ranging from 2 h to overnight (or <30 min when combined with Ctip2 immunostaining), the brain was cut into 20-μm sections. Retrogradely labeled cells were examined in M2 (oblique or sagittal sections) from OFC and PPC, and in OFC (sagittal or coronal sections), PPC (sagittal or coronal sections), and the perirhinal cortex (PRC; coronal sections) from M2. Every four serial sections were collected as a set. The first or third section of each set was used to count labeled cells. The remaining sections were used for the determination of cortical areas and layers.

For area identification, immunostaining for NF-H and NeuN was used. Adjacent sections were incubated overnight at 4°C with a mouse monoclonal antibody against NF-H (N-200 antibody, N0142, Sigma-Aldrich Co. LLC, St. Louis, MO, USA; 1:1000) or a mouse monoclonal antibody against NeuN (1:5000) and a guinea pig polyclonal antibody against VGluT2 (1:5000). After washes with TBS, the sections were incubated with an Alexa Fluor-conjugated secondary antibody (1:200).

To visualize Ctip2 immunoreactivity in retrogradely labeled cells, sections were incubated overnight at 4°C with a rat monoclonal antibody against Ctip2 (1:500) in 0.05 M TBS containing 10% normal goat serum, 2% bovine serum albumin, and 0.5% Triton X-100. After washing in TBS, sections were reacted with an Alexa Fluor 488-conjugated secondary antibody (1:200).

Injection coordinates of M2 were 4–4.5 mm anterior to bregma and 1–2 mm lateral to the midline at four depths (0.2, 0.4, 0.6, and 0.8 mm from the surface, with 25° rostral inclination of pipettes). Injection areas of OFC (including lateral orbital and dorsolateral orbital areas) were 5 mm anterior to bregma, 2–3 mm lateral, and 2–3 mm deep (with a 25° rostral inclination of pipettes), and those for PPC were 3.5 mm posterior to bregma and 2–3 mm lateral at three depths (0.2, 0.4, and 0.6 mm from the surface).

In some cases of CTB555 injection into OFC or M1, Fast Blue was also injected into PRC to examine double-labeling of M2 cells projecting to PRC and those projecting to OFC or M1. The injection approach to the PRC area was described previously (Hirai et al., 2012). In brief, the injection pipette was advanced with a 30° lateral inclination using positions of blood vessels and the rhinal sulcus as a reference.

### ANTEROGRADE LABELING OF CC FIBERS

Biotinylated dextran amine (10% w/v in 0.5 M potassium acetate; BDA-10K; Life Technologies Corporation) was injected into L1 to L5 of M2 by pressure injection from glass micropipettes (tip diameter, 50–100 μm). After a survival period of 7–10 days, the animals were deeply anesthetized with sodium pentobarbital and perfused with a prefixative followed by a fixative (4% paraformaldehyde and 0.2% picric acid in 0.1 M PB). The brain was cut into 50-μm coronal sections using a vibratome.

BDA-10K was visualized by incubating sections with avidin–biotin–peroxidase complex (1%; ABC Elite, Vector Laboratories, Inc., Burlingame, CA, USA) in 0.05 M TBS overnight at 4°C. To enhance the signal, sections were reacted for 30 min at room temperature with 2.5 μM biotinylated tyramine, 3 μg/mL glucose oxidase, and 2 mg/mL β-D-glucose in 2% bovine serum albumin, which was dissolved in 0.05 M Tris-buffered (TB) solution. Sections were subsequently incubated with Alexa Fluor 488-conjugated streptavidin (Life Technologies Corporation; 1:200) for 2–3 h at room temperature. Next, the same sections were incubated overnight at 4°C with a guinea pig polyclonal antibody against VGluT2 (1:5000) and a mouse monoclonal antibody against NeuN (1:5000). After washes with TBS, the sections were incubated with secondary antibodies conjugated to Alexa Fluor 594 (for VGluT2) and Alexa Fluor 350 (for NeuN) for 2–3 h at room temperature. For area identification, adjacent sections were incubated with N-200 antibody (1:1000).

To evaluate the laminar distribution of CC innervations from M2, RGB color images were collected using a DP73 microscope camera (Olympus Corporation, Tokyo, Japan), converted to gray-scale, and analyzed with Image J software. The density of anterogradely labeled fibers was measured in L1 to L5 with a width of 0.1 mm in each target area of M2 to obtain the laminar distribution index, [(fiber density in L1) - (fiber density in L2/3)]/[(fiber density in L1) + (fiber density in L2/3)]. The laminar distributions of anterogradely labeled fibers from M2 were examined in OFC, M1, PRC 36, PPC, and contralateral M2.

### *IN VITRO* ELECTROPHYSIOLOGICAL RECORDINGS OF RETROGRADELY LABELED CELLS

Rats (postnatal days 17–21) were anesthetized with a mixture of ketamine (40 mg/kg, i.p.) and xylazine (4 mg/kg, i.p.) and placed in a stereotaxic apparatus. For simultaneous labeling of COM cells and PRC-projecting cells, green fluorescent Retrobeads (Lumafluor, Inc., Durham, NC, USA) and CTB555 were injected into contralateral M2 and ipsilateral PRC, respectively. To label corticothalamic (CTh) cells, CTB555 was injected into the ipsilateral ventral thalamic nuclei. One or two days after tracer injection (postnatal days 19–23), animals were deeply anesthetized with isoflurane and decapitated. The brain was quickly removed and submerged in ice-cold physiological Ringer’s solution. Six 300-μm-thick slices were obtained from M2 ipsilateral to the PRC or thalamic injection site. Slices were immersed in a buffered solution containing 125 mM NaCl, 2.5 mM KCl, 2 mM CaCl_2_, 1 mM MgCl_2_, 25 mM NaHCO_3_, 1.25 mM NaH_2_PO_4_, 10 mM glucose, and 4 mM lactic acid. This solution was continuously bubbled with a mixture of 95% O_2_ and 5% CO_2_. Lactic acid was omitted during recordings. In some recordings from CTh cells (13/53 cells), glutamatergic synaptic transmission was blocked by supplemental application of 50 μM D-(-)-2-amino-5-phosphonopentanoic acid (D-AP5; R & D Systems, Inc., Minneapolis, MN, USA) and 20 μM 6-cyano-7-nitro-quinoxaline-2,3-dione (CNQX; Funakoshi, Tokyo, Japan), and GABA_A_ receptors were blocked with 50 μM picrotoxin (Sigma-Aldrich Co. LLC). The recordings were made in whole-cell mode at 30–31°C. Labeled cells were identified using epifluorescence microscopy (BX50WI, Olympus Corporation) with a 40× water-immersion objective (numerical aperture = 0.8, Olympus Corporation).

The pipette solution for current-clamp recording consisted of 130 mM potassium methylsulfate, 0.5 mM EGTA, 2 mM MgCl_2_, 2 mM Na_2_ATP, 0.2 mM GTP, and 20 mM HEPES, with 0.75% biocytin. The pH of the solution was adjusted to 7.2 using KOH, and the osmolarity was 290 mOsm. The membrane potentials were not corrected for liquid junction potentials. The series resistance of the recording cells was <25 MΩ. The firing responses to depolarizing current pulses were recorded within 5 min from whole-cell break-in. Recordings were amplified with a Multiclamp 700B amplifier (Molecular Devices, LLC, Sunnyvale, CA, USA), digitized at 10 kHz using a Digidata 1440A apparatus (Molecular Devices, LLC), and collected with pClamp 10 software (Molecular Devices, LLC). Data were analyzed with IGOR Pro software (WaveMetrics, Inc., Lake Oswego, OR, USA), including NeuroMatic functions^2^.

### CORTICAL AREA IDENTIFICATION

To identify individual cortical areas and to confirm the injection localization to those areas, the following criteria were used.

#### Frontal areas

N-200 staining of L2/3 to upper L5 in M2 was weaker than that in M1 or that in OFC (Ueta et al., 2013). However, staining in M2 was stronger than that in the anterior cingulate area. Subdivisions of OFC were identified by cytoarchitecture and N-200 staining (Van De Werd and Uylings, 2008). M2 was intimately connected with the lateral part (weaker in N-200 staining) of the lateral orbital and dorsolateral orbital areas in OFC. These laminar structures were determined in a similar manner to M2.

#### PRC

The areal and laminar structures of area 36 (PRC 36) and area 35 (PRC 35) were identified by immunostaining for N-200 (stronger staining at superficial layers in PRC 36 than PRC 35; Hirai et al., 2012), VGluT2 [stronger staining at layer 4 (L4) or lower at L2/3 in PRC 36 than PRC 35], Ctip2 [positive cells distributed mainly in L5 and layer 6 (L6) of PRC 36, but also in L2/3 of PRC 35], or NeuN (L4 found in PRC 36, but not in PRC 35).

#### PPC

The PPC area is situated just caudal to the M1 hindlimb area, and rostral to both the secondary visual cortex and retrosplenial cortex. PPC demonstrated stronger NF-H staining than the adjacent caudal cortical areas. The border between L2/3 and L5 was determined by VGluT2 immunoreactivity (stronger in L2/3) and pan-neuronal staining. Localization of retrograde tracer deposition to PPC was confirmed by differences in retrograde labeling among thalamic nuclei: labeled cells were abundant in the LP nucleus after PPC injection, and abundant in the ventral anterior/ventromedial, ventrolateral, and posterior thalamic nuclei upon adjacent M1 injection (Reep et al., 1994). Thalamic nuclei were identified by calbindin expression pattern in addition to pan-neuronal staining, as reported previously (Ushimaru et al., 2012). Abbreviations used in this paper are summarized in **Table 1**.

**Table 1:** Abbreviations for brain areas, projection types, and firing types of pyramidal cells.

Abbreviations	Description
**Cortical areas**
M1	Primary motor cortex
M2	Secondary motor cortex
OFC	Orbitofrontal cortex
Pir	Piriform cortex
PPC	Posterior parietal cortex
PRC	Perirhinal cortex
Te	Ventral temporal association cortex
**Thalamic nuclei**
LP	Lateral posterior nucleus
Po	Posterior nucleus
VA	Ventral anterior nucleus
VL	Ventrolateral nucleus
VM	Ventromedial nucleus
**Projection types**
CCS	Crossed corticostriatal
COM	Commissural
CPn	Corticopontine
CSp	Corticospinal
CTc	Corticotectal
CTh	Corticothalamic
CC	Corticocortical
iCC	Ipsilateral corticocortical
**Firing types**
FA	Fast adapting
SA	Slow adapting
SA-d	Slow adapting with initial doublet

### QUANTITATIVE ANALYSIS OF AXON MORPHOLOGIES

Axon varicosities of biocytin-labeled L5 CTh and PRC-projecting cells (preparations obtained from Hirai et al., 2012) were measured with 100× objective combined with a further 1.25× magnification, using the Neurolucida system (MBF bioscience, Williston, VT, USA) and analyzed quantitatively with NeuroExplorer software (MBF bioscience) and IGOR Pro software. Axon varicosities were defined as darkly stained axonal dilations, typically about 1.5-fold wider than adjoining fibers. Serial images (0.5-μm step depth) of axon varicosities were acquired by the Neurolucida system, and were stacked into Adobe Photoshop software (Adobe Systems, Mountain View, CA, USA).

### STATISTICS

Data are presented as the mean ± standard deviation (SD). Pairwise data on the proportion of Ctip2-positive cells among iCC cells were compared with the chi-square test. The ratio of Ctip2-positive and Ctip2-negative cells was compared to an even split (50%) in individual projections with a one-sample *t*-test. The Mann–Whitney *U*-test was used for two-group comparisons. The difference of axon length and varicosity distributions within L1 and L2/3 was tested by Kolmogorov–Smirnov two-sample test. To assess the difference in anterogradely labeled fiber density between L1 and L2/3, a two-tailed one-sample *t*-test was used to compare the laminar distribution index to a no-difference value (index = 0). The Tukey–Kramer multiple comparisons test was used for statistical comparisons of the L1 sublaminar distribution patterns of anterogradely labeled fibers in OFC, M1, PRC 36, PPC, and contralateral M2. Significance was set at *P* values <0.05.

## RESULTS

### MULTIPLE PYRAMIDAL CELL SUBTYPES IN THE RAT FRONTAL CORTEX

**FIGURE 1:**
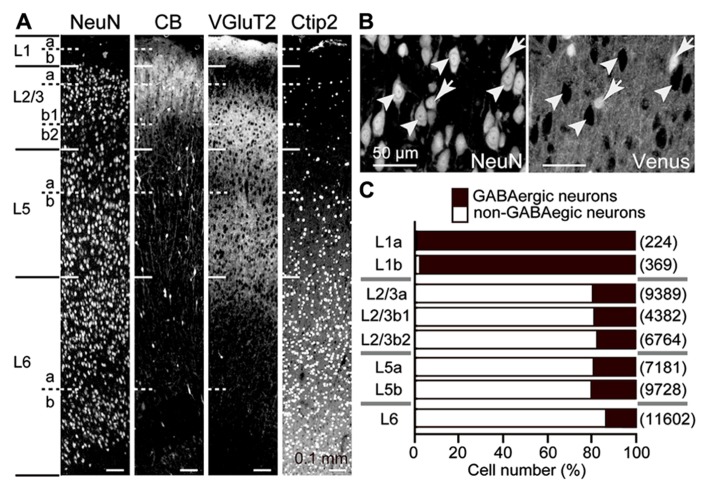
**GABAergic and non-GABAergic neuronal populations in M2 sublaminae.** **(A)** Laminar identification of the M2 area by immunofluorescence for NeuN, calbindin (CB), VGluT2, and Ctip2. L1, L2/3, L5, and L6 were identified by cytoarchitecture. L1a demonstrates higher immunoreactivity for VGluT2 than L1b. L2/3a demonstrates weaker immunoreactivity for VGluT2 than L2/3b. L2/3b1 is immunopositive for CB, but L2/3b2 is not. L5 and L6 demonstrate higher immunoreactivity for Ctip2 than superficial layers. L5a demonstrates weaker immunoreactivity for VGluT2 and Ctip2 than L5b. L6a and L6b are divided by an intervening neuron-sparse zone. Oblique section, 20 μm thickness. **(B)** Identification of GABAergic and non-GABAergic neurons. Non-GABAergic neurons were identified by NeuN expression without Venus expression (arrowheads) in the M2 area of VGAT-Venus rats, which express fluorescent protein Venus in GABAergic neurons (arrows). Oblique section, 8 μm thickness. **(C)** The proportion of GABAergic and non-GABAergic neurons in each sublayer. Black bar, GABAergic neurons; white bar, non-GABAergic neurons. (*n*), total number of counted neurons.

The rat frontal cortical layers can be further divided into several sublayers by the size and density of neuronal somata, calbindin expression, thalamic fiber density, and Ctip2 expression (**Figure 1A**; Ueta et al., 2013). In all cortical layers below L1, about 80% of neurons were non-GABAergic, mostly pyramidal cells (**Figures 1B,C**). Among pyramidal cells, those in L5, which provide the major outputs of the cortex to subcortical and subcerebral areas, are highly differentiated in their physiological and morphological characteristics and intralaminar and interlaminar connectivities (Morishima and Kawaguchi, 2006; Hattox and Nelson, 2007; Otsuka and Kawaguchi, 2008, 2011; Brown and Hestrin, 2009; Morishima et al., 2011; Avesar and Gulledge, 2012; Hirai et al., 2012; Ueta et al., 2013).

**FIGURE 2:**
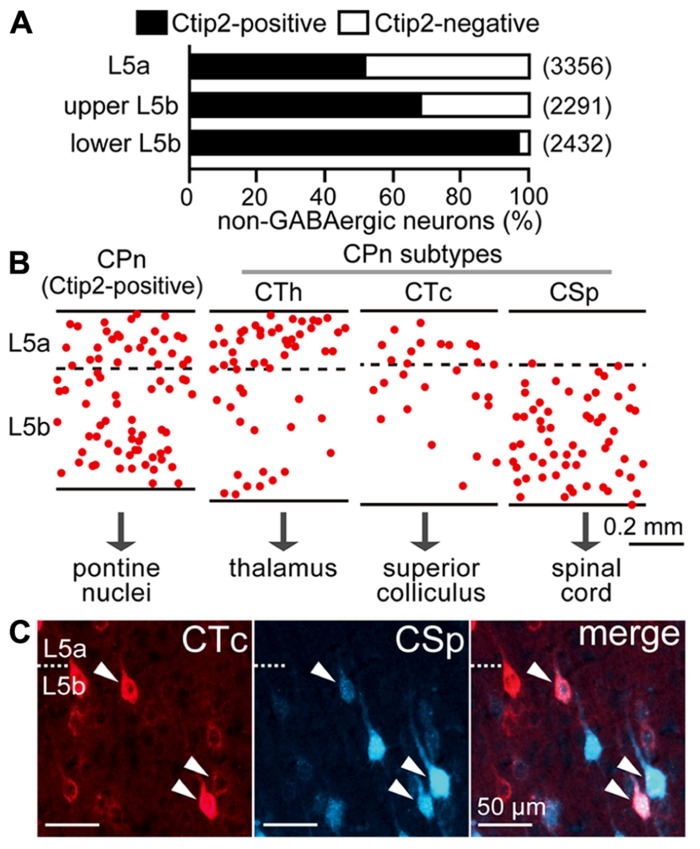
**Diversity of pyramidal cell subtypes dependent on L5 depth.** **(A)** The proportions of Ctip2-positive and Ctip2-negative cells dependent on L5 depth. NeuN-positive and Venus-negative populations (corresponding to non-GABAergic neurons) were identified in the M2 area of VGAT-Venus rats. L5b was divided into upper and lower halves. Black bar, Ctip2-positive cells; white bar, Ctip2-negative cells. (*n*), total number of counted neurons. **(B)** Comparative laminar distributions of CPn cell subtypes in M2 L5: corticothalamic (CTh), corticotectal (CTc), and corticospinal (CSp) cells. CPn cells were retrogradely labeled by Fast Blue. Each dot corresponds to a single retrogradely labeled neuron. Oblique section, 20 μm thickness. **(C)** Most L5b CTc cells were also labeled from the spinal cord (arrowheads). CTc and CSp cells were labeled by CTB555 and Fast Blue in the same animal, respectively. Oblique section, 20 μm thickness.

L5 pyramidal cells consist primarily of Ctip2-positive CPn cells and Ctip2-negative COM cells (Arlotta et al., 2005; Ueta et al., 2013). The proportion of these two major subtypes in the M2 area changed according to depth within L5: both subtypes were abundant in L5a and upper L5b, but in lower L5b, Ctip2-positive cells predominated, suggesting that CPn cells are more prevalent in the lower half of L5b (**Figure 2A**). Furthermore, both CPn and COM cells comprise a variety of subtypes, as described below.

Corticopontine cells innervate additional subcortical and subcerebral targets, including the thalamus, superior colliculus, and spinal cord (**Figure 2B**), relating to their depth within L5. The distribution of corticospinal (CSp) cells is restricted to L5b, whereas CTh cells projecting to ventral thalamic nuclei in L5a are more abundant than those in L5b (Hirai et al., 2012; Ueta et al., 2013). L5b CTh cells send axons to the spinal cord (Ueta et al., 2013). Corticotectal (CTc) cells projecting to intermediate and deep zones of the superior colliculus were commonly found at the border between L5a and L5b, and L5b CTc cells also sent axons to the spinal cord (**Figure 2C**).

Similarly, COM cells are differentiated into two subtypes according to their projections to the striatum (Otsuka and Kawaguchi, 2011). One subtype (COM type I) projects to the contralateral cortex and ipsilateral striatum, while another subtype (COM type II) additionally projects to the contralateral striatum [crossed corticostriatal (CCS) cells; Wilson, 1987; Reiner et al., 2003; Morishima and Kawaguchi, 2006]. These results indicate that L5 of the rat frontal cortex contains multiple CPn and COM cell subtypes that differ in their long-distance axon collateralizations.

### IMPLICATION FOR ICC CONNECTIONAL ORGANIZATION FROM THE M2 TO M1 PROJECTION PATTERN

**FIGURE 3:**
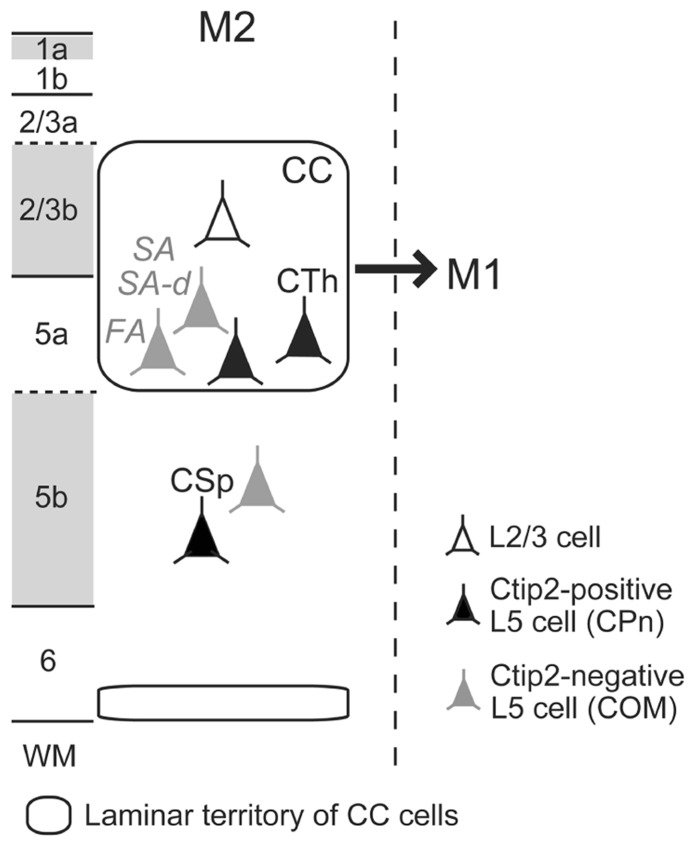
**Scheme of ipsilateral corticocortical (iCC) projections from M2 to M1.** Multiple L5 CPn (filled-black) and COM (filled-gray) cell subtypes participate in iCC projection from M2 to M1. L1a, L2/3b, and L5b receive dense thalamocortical inputs, labeled by VGluT2 immunoreactivity (gray). FA, fast adapting; SA, slow adapting; SA-d, slow adapting with initial doublet firing; WM, white matter.

We recently found that iCC projections to M1 preferentially originate from lower L2/3 (L2/3b) and L5a of M2 (**Figure 3**; Ueta et al., 2013). Both CPn cells and two subtypes of COM cells in L5a of M2 send axons to M1. L5a CTh cells in M2 innervate upper L1 (L1a) of M1 (Ueta et al., 2013), similar to their local projections to L1a within M2 (Hirai et al., 2012). Between visual cortical areas, L1 innervation is denser in the direction from higher to lower areas [called “feedback (top-down) connections”] than in the opposite direction [“feedforward (bottom-up) connections”; Coogan and Burkhalter, 1993; Dong et al., 2004]. Analogous to the visual system, we observed that the iCC projection from M2 to M1 forms a top-down type of anatomical connectivity (Ueta et al., 2013). This result gives rise to the hypothesis that the participation of certain pyramidal cell subtypes, especially those of L5, in reciprocal iCC connections correlates with the functional relationship between the two areas.

M2 projects to various ipsilateral cortices, located proximally or distally. To develop a more generalized organization scheme of M2 iCC connectivity, we compared the iCC connections of M2 with a caudally situated adjacent frontal area (M1), a rostral adjacent area (OFC), a distant polysensory area (PPC), and distant declarative memory-related areas (PRC 36 and PRC 35) by investigating the laminar distributions of iCC cells in the source area, their innervations in the target area, and the composition of L5 pyramidal cell subtypes participating in these iCC projections.

### LAMINAR DISTRIBUTIONS OF iCC CELLS IN RECIPROCAL CONNECTIONS OF M2 AND ADJACENT FRONTAL AREAS

**FIGURE 4:**
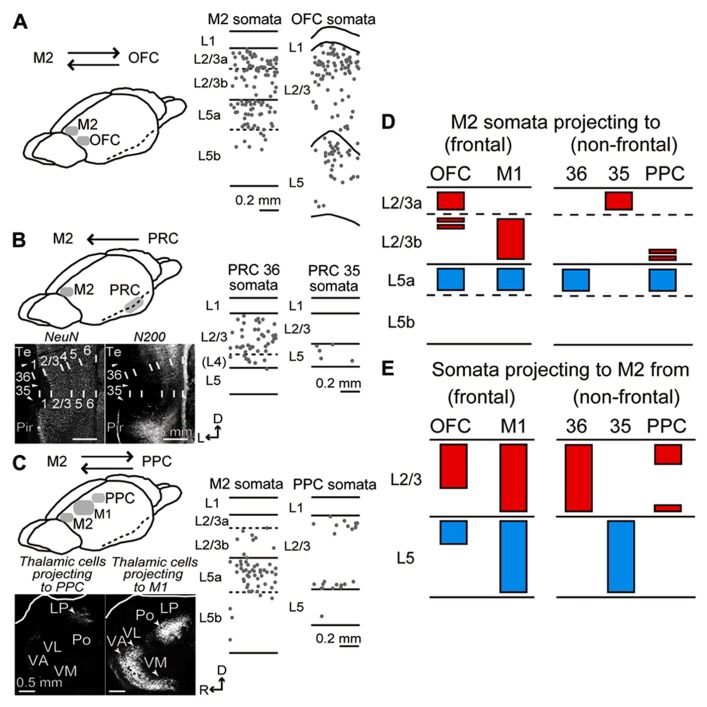
**Laminar distributions of iCC cells connecting M2 to OFC, PRC, and PPC.** **(A)** iCC connections between M2 and OFC. Laminar distributions of M2 cells retrogradely labeled by CTB555 from OFC (middle), and OFC cells labeled from M2 (right). In OFC, CTB555 was injected into lateral orbital and dorsolateral orbital areas. iCC cells were mainly distributed in upper L2/3 and upper L5 in both areas. Each dot represents a single retrogradely labeled neuron. Sagittal section, 50 μm thickness. **(B)** iCC connections between M2 and PRC. Bottom left, PRC area identification by immunostaining for NeuN and NF-H (N-200 antibody). Note the N-200 staining differences in the superficial layers among cortical areas. Arrowhead, area border. 35, area 35 of PRC (PRC 35); 36, area 36 of PRC (PRC 36); pir, piriform cortex; Te, ventral temporal association cortex. D, dorsal; L, lateral. Coronal section, 50 μm thickness. Middle and right, laminar distributions of PRC 36 and PRC 35 cells retrogradely labeled by CTB555 from M2. Note iCC cells in L2/3 of PRC 36, but in L5 of PRC 35. **(C)** iCC connections between M2 and PPC. Bottom left, retrograde tracer injection into PPC-labeled thalamic cells in lateral posterior (LP) nucleus, while injection into the rostrally adjacent M1-labeled thalamic cells in ventral anterior/ventromedial (VA/VM), ventrolateral (VL), and posterior (Po) nuclei. Sagittal section, 50 μm thickness. Middle and right, laminar distributions of M2 cells retrogradely labeled by CTB555 from PPC, and PPC cells labeled by CTB555 from M2. PPC-projecting cells were mainly distributed in L5a of M2, whereas M2-projecting cells labeled the superficial and bottom parts of L2/3 of PPC. D, dorsal; R, rostral. **(D)** Laminar patterns of M2 somata projecting to the adjacent frontal areas (OFC and M1) and distant non-frontal areas (PRC 36, PRC 35, and PPC). Red, somata distribution in L2/3; blue, distribution in L5. Based on the present data combined with Hirai et al. (2012) (PRC) and Ueta et al. (2013) (M1). **(E)** Laminar patterns of somata projecting from the adjacent and distant areas to M2. Based on the present data combined with Ueta et al. (2013) (M1).

We found that the laminar distributions of iCC cells projecting to adjacent frontal areas were more similar between M2 and OFC than between M2 and M1. Both M2 cells projecting to OFC and OFC cells projecting to M2 mainly originated from upper L2/3 and upper L5 (**Figure 4A**; three rats per analysis). By contrast, between M2 and M1, M1 iCC cells were distributed widely from L2/3a to L6b, whereas M2 iCC cells, mainly distributed from L2/3b and L5a, were more restricted in territory (Ueta et al., 2013).

### LAMINAR DISTRIBUTIONS OF iCC CELLS CONNECTING M2 AND DISTANT NON-FRONTAL AREAS

We found that the laminar distributions of iCC cells connecting M2 and non-frontal distant areas were strongly related to the combination of source and target areas. PRC 36 cells projecting to M2 were observed in superficial layers (L2/3 and L4) and L6, but rarely in L5 (**Figure 4B**, middle; three rats), whereas M2 cells projecting to PRC 36 were distributed mainly in L5a (Hirai et al., 2012). By contrast, PRC 35 cells projecting to M2 were found exclusively, but in reduced numbers, in L5 (**Figure 4B**, right; three rats), whereas M2 cells projecting to PRC 35 were distributed mainly in L2/3a (Hirai et al., 2012). In PPC, M2-projecting cells were mainly located at the upper and bottom portions of L2/3 and deep L6 (**Figure 4C**, right). In M2, on the other hand, PPC-projecting cells were mainly localized to L5a (**Figure 4C**, middle) and situated in the medial part of M2 (data not shown).

Therefore, between M2 and the adjacent cortical areas, both L2/3 and L5 cells participate in both directions of reciprocal connections (**Figures 4D**, left; **4E**, left), whereas, between M2 and the distant areas, either L2/3 or L5 cells participate in one direction (**Figures 4D**, right; **4E**, right). Additionally, pyramidal cells in M2 L5a projected to all the adjacent and distant cortical areas assessed with the exception of PRC 35 (**Figure 4D**).

### PARTICIPATION OF L5 PYRAMIDAL CELL SUBTYPES IN iCC PROJECTIONS IS RELATED TO BOTH THE SOURCE AND TARGET AREAS

**Figure 5:**
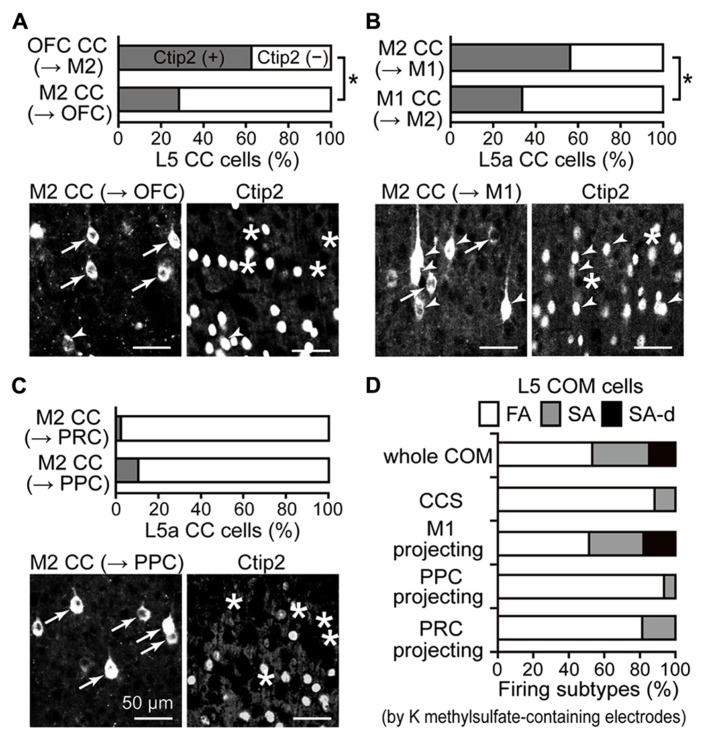
**iCC projection-specific participation of Ctip2-positive CPn cells.** **(A)** Ctip2 expression in OFC upper L5 cells projecting to M2 and in M2 L5a cells projecting to OFC. Upper graph, the proportion of Ctip2-positive cells was higher among OFC cells projecting to M2 than M2 cells projecting in the opposite direction (*P* < 0.01, chi-square test). OFC cells projecting to M2 similarly contained Ctip2-positive and Ctip2-negative cells (*P* = 0.29, one-sample *t*-test), but M2 cells projecting to OFC contain more Ctip2-negative cells than Ctip2-positive cells (*P* < 0.05). Lower photograph, M2 cells projecting to OFC (left, labeled by CTB555), which contain cells positive for Ctip2 (arrowheads in left and right) and negative for Ctip2 (arrows in left, asterisks in right). **(B)** Ctip2 expression in M2 L5a cells projecting to M1 and in M1 L5a cells projecting to M2. Upper graph, the proportion of Ctip2-positive cells was higher among M2 cells projecting to M1 than M1 cells projecting in the opposite direction (*P* < 0.01, chi-square test). M2 cells projecting to M1 similarly contained Ctip2-positive and Ctip2-negative cells (*P* = 0.68, one-sample *t*-test), but M1 cells projecting to M2 contain more Ctip2-negative cells than Ctip2-positive cells (*P* < 0.01). Data taken from Ueta et al. (2013). Lower photograph, M2 cells projecting to M1, which contain cells positive for Ctip2 (arrowheads) and negative for Ctip2 (arrows in left, asterisks in right). **(C)** Ctip2 expression in M2 L5a cells projecting to PRC and to PPC. Upper graph, the proportion of Ctip2-positive cells among M2 L5a cells projecting to distant non-frontal areas. M2 L5a cells projecting to PRC and PPC both contain more Ctip2-negative cells than Ctip2-positive cells (*P* < 0.01, respectively, one-sample *t*-test), and demonstrate lower proportion of Ctip2-positive cells than M2 L5a cells projecting to frontal areas (OFC and M1; *P* < 0.01, chi-square test). Lower photograph, M2 cells projecting to PPC negative for Ctip2 (arrows in left, asterisks in right). **(D)** Proportion of firing subtypes among L5 COM cell subtypes in M2, identified by retrograde labeling. COM cells consist of all three firing types (53.1, 32, and 14.8% for FA, SA, and SA-d types; *n* = 431). CCS cells, a type of COM cell, consist mostly of FA-type cells, but contain no SA-d-type cells (88.2, 11.8, and 0% for FA, SA, and SA-d types; *n* = 34). Similar to the entire COM cell population, COM cells projecting to M1 consist of all three firing types (51.3, 30.8, and 17.9%, for FA, SA, and SA-d types; *n* = 39). By contrast, COM cells projecting to PPC (93.5, 6.5, and 0% for FA, SA, and SA-d types; *n* = 31) or to PRC (81.3, 18.8, and 0% for FA, SA, and SA-d types; *n* = 32), like CCS cells, consist mostly of FA-type cells and contain no SA-d-type cells. Data, except for COM cells projecting to PRC, are taken from Otsuka and Kawaguchi (2008, 2011) and Ueta et al. (2013).

Corticopontine and COM cell subtypes in L5 are distinguished by Ctip2 molecular expression (Arlotta et al., 2005; Ueta et al., 2013). We examined the Ctip2 expression pattern in individual iCC projections originating from L5 and found that ratios of Ctip2-positive CPn cells and Ctip2-negative COM cells systematically differed according to the iCC projection type. The fraction of Ctip2-positive cells in L5 was higher in connections in the direction from OFC to M2 (62.5% in L5; *n* = 304 cells, three rats) than in connections directed from M2 to OFC (**Figure 5A**; 28.4% in L5a; *n* = 338, three rats; *P* < 0.01, chi-square test). As reported previously, the fraction of Ctip2-positive cells in L5a was higher in connections in the direction from M2 to M1 (56.3% in L5a; *n* = 679, four rats) than in connections directed from M1 to M2 (**Figure 5B**; 33.8% in L5a; *n* = 225, three rats; *P* < 0.01, chi-square test; data from Ueta et al., 2013). We found that iCC projections to distant areas that originated from M2 L5a consisted mainly of Ctip2-negative cells (**Figure 5C**; Ctip2-positive cell ratio: M2 to PRC projection, 2.6%, *n* = 823, three rats; M2 to PPC projection, 10.4%, *n* = 259, three rats). Therefore, L5 CPn and COM cells in the source area, especially M2, participate differently in iCC projection based on the target area.

COM cells demonstrate heterogeneous firing patterns as assessed by depolarized somatic current injection: a slow adapting (SA) type, a SA type with an initial doublet firing (SA-d), and a fast adapting (FA) type (**Figure 5D**, whole COM; Otsuka and Kawaguchi, 2008). All three firing types were observed simultaneously among L5 COM cells projecting to M1 (**Figure 5D**, M1-projecting; Ueta et al., 2013), but those projecting to PPC demonstrated more FA-type firing, similar to CCS cells innervating both sides of striatum in addition to the contralateral cortex (**Figure 5D**, CCS and PPC-projecting; Otsuka and Kawaguchi, 2008, 2011). We further investigated the firing subtype composition of COM cells projecting to PRC.

We found that the L5a COM cell population projecting to PRC contained more FA-type cells (**Figure 5D**, PRC-projecting), whereas L5a CTh cells, a CPn subtype in L5a, demonstrated more SA- or SA-d-type cells. We divided retrogradely labeled L5a cells (32 cells simultaneously from PRC and the contralateral M2; 53 cells from the thalamus; 36 cells from contralateral M2 in rats without other injections) into three classes using two firing parameters obtained from interspike intervals (ISIs) during the current pulse injection (recorded with potassium methylsulfate solution; fixed amplitude, 0.5 nA; duration, 1 s): (1) the firing frequencies calculated from the first ISIs (f1) and (2) the ratio of firing frequencies calculated from the seventh and second ISIs (f7/f2; adaptation index). We classified cells with f7/f2 < 0.5 as FA type (81.3% of PRC-projecting COM cells; no FA-type CTh cells; 55.6% of L5a COM cells), cells with f7/f2 > 0.5 as SA type (18.8% of PRC-projecting COM cells; 9.4% of CTh cells; 33.3% of L5a COM cells), and SA cells with f1 > 80 Hz as SA-d type (no SA-d type PRC-projecting COM cells; 90.6% of CTh cells; 11.1% of L5a COM cells). Therefore, COM cell subtypes differentially participate in iCC projection depending on the target area.

### LAMINAR DISTRIBUTIONS OF FIBERS FROM M2 IN IPSILATERAL CORTICAL AREAS AND CONTRALATERAL M2

**FIGURE 6:**
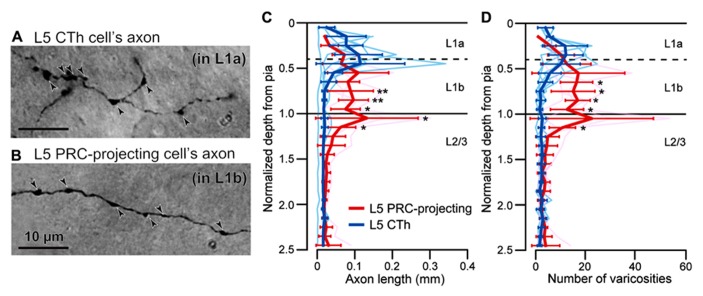
**Correlation of axonal length and varicosity number of superficial layer branches from L5 pyramidal cells. (A)** L1a axon varicosities (arrowheads) of an L5a CTh cell. **(B)** L1b axon varicosities (arrowheads) of an L5a PRC-projecting cell. **(C)** Axon length distributions of CTh cells (blue, six branches of two cells) and PRC-projecting cells (red, five branches of two cells) within L1 and L2/3. Cortical depth was normalized by L1 thickness: 0, cortical surface; 1, L1/L2 border. Axon length and varicosity number were measured in each depth fraction (fraction length, one-tenth of L1 thickness). Thin lines, individual axon branches; thick lines, mean ± SD. Asterisks indicate significant differences between L5 PRC-projecting and CTh cells in each bin (*P < 0.05, **P < 0.01; Mann–Whitney *U*-test). **(D)** Axon varicosity distributions of CTh cells (blue) and L5 PRC-projecting cells (red). *P < 0.05, Mann—Whitney *U*-test.

To characterize innervation patterns from M2 to target areas, we quantified the relative laminar density of axon fibers in individual areas. First, we confirmed correlation of axonal length and the frequency of their varicosities, most of which correspond to synaptic boutons (**Figures 6A,B**; Kisvárday et al., 1986; Gabbott et al., 1987). Previously we found that L5a CTh cells distribute axon collaterals in the upper part of L1 than PRC-projecting cells (**Figure 6C**; Hirai et al., 2012). Similar to the axon length distributions, we found that axon varicosities of CTh cell were found more in the upper part of L1, whereas those of PRC-projecting cell more in the lower part of L1 (**Figure 6D**). No differences were found between the distributions of axon lengths and varicosities along the depth in both subtypes (Kolmogorov–Smirnov two-sample test). The axon length of individual branches correlated linearly with the number of their axon varicosities in both subtypes (CTh cells, correlation coefficient = 0.87 ± 0.05, six branches of two cells; PRC-projecting cells, correlation coefficient = 0.83 ± 0.29, five branches of two cells).

Next, to examine innervation patterns of M2 in its iCC target areas, we injected the anterograde tracer BDA-10K in M2. We found that the laminar distribution pattern of labeled fibers differed among target areas of M2. To quantify innervation preference for L1 or L2/3 of target areas, we normalized the fiber density to the maximum value (max = 1) in each section and obtained a laminar distribution index from +1 (totally L1) to -1 (totally L2/3), with 0 indicating no difference between L1 and L2/3 (see Materials and Methods). To examine the innervation preference within L1, we divided L1 into four parts (upper and lower halves of L1a and L1b, respectively) and compared the density across L1 subdivisions.

**Figure 7:**
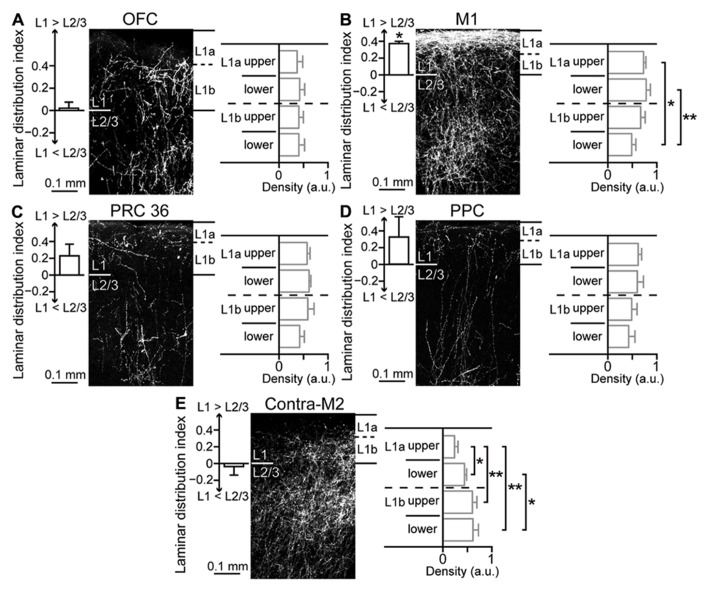
**Laminar pattern of fiber terminations arising from M2 to ipsilateral cortical areas and contralateral M2. (A)** M2-derived fiber distributions in L1 and L2/3 of ipsilateral OFC, labeled with BDA-10K injections into L1 to L5 of M2. Left graph, uniform fiber distribution between L1 and L2/3 (*P* = 0.5, two-tailed one-sample *t*-test). Laminar distribution index, [(fiber density in L1) - (fiber density in L2/3)]/[(fiber density in L1) + (fiber density in L2/3)], is positive for L1 preference and negative for L2/3 preference. Right graph, uniform fiber distributions along L1, determined by comparing four subdivisions (upper and lower halves of L1a and L1b, respectively). **(B)** M2-derived fiber distributions in ipsilateral M1. Left graph, denser distribution in L1 than in L2/3 (*P* < 0.01). Right graph, denser distribution in L1a than in L1b (*P < 0.05, **P < 0.01; Tukey–Kramer multiple comparisons test). Data taken from Ueta et al. (2013). **(C)** M2- derived fiber distributions in ipsilateral PRC 36. Left graph, a non-significant trend was observed for denser distribution in L1 than in L2/3 (*P* = 0.1). Right graph, uniform distributions along L1. **(D)** M2-derived fiber distributions in ipsilateral PPC, similar to that in ipsilateral PRC 36 (*P* = 0.15). **(E)** M2-derived fiber distributions in contralateral M2. Left graph, uniform distribution between L1 and L2/3 (*P* = 0.53). Right graph, denser distribution in L1b than in L1a (*P < 0.05, **P < 0.01; Tukey-Kramer multiple comparisons test).

Labeled fibers in OFC were similarly distributed in L1 and L2/3 (**Figure 7A**, left; laminar distribution index, 0.02 ± 0.05, four rats) and localized uniformly within L1 (**Figure 7A**, right; fiber density, 0.37 ± 0.12 in upper L1a, 0.42 ± 0.1 in lower L1a, 0.4 ± 0.09 in upper L1b, and 0.41 ± 0.12 in lower L1b). As reported previously, labeled fibers in M1 were more abundant in L1 than in L2/3 (**Figure 7B**, left; data from Ueta et al., 2013; laminar distribution index, 0.37 ± 0.03, three rats; P < 0.05, two-tailed one-sample *t*-test). Within L1, labeled fibers in L1a were more abundant than those in L1b (**Figure 7B**, right; 0.73 ± 0.05 in upper L1a, 0.78 ± 0.09 in lower L1a, 0.67 ± 0.09 in upper L1b, and 0.49 ± 0.08 in lower L1b; *P < 0.05, **P < 0.01, Tukey–Kramer multiple comparisons test).

Labeled fibers seemed to demonstrate preference for L1 over L2/3 in the distant areas PRC 36 (**Figure 7C**, left; laminar distribution index, 0.23 ± 0.14, three rats) and PPC (**Figure 7D**, left; laminar distribution index, 0.32 ± 0.24, three rats). The L1 innervation pattern was similar between PRC 36 (**Figure 7C**, right; 0.57 ± 0.06 in upper L1a, 0.61 ± 0.04 in lower L1a, 0.59 ± 0.13 in upper L1b, and 0.42 ± 0.09 in lower L1b) and PPC (**Figure 7D**, right; 0.62 ± 0.07 in upper L1a, 0.6 ± 0.13 in lower L1a, 0.49 ± 0.11 in upper L1b, and 0.43 ± 0.13 in lower L1b).

Callosal fibers issue from COM cells, but not from CPn cells. We found that labeled fibers in contralateral M2 were abundant in both L1 and L2/3 (**Figure 7E**, left; laminar distribution index, -0.04 ± 0.1, four rats). Within L1, however, labeled fibers in L1b were more abundant than those in L1a (**Figure 7E**, right; 0.24 ± 0.07 in upper L1a, 0.44 ± 0.04 in lower L1a, 0.6 ± 0.09 in upper L1b, and 0.61 ± 0.11 in lower L1b; *P < 0.05, **P < 0.01, Tukey–Kramer multiple comparisons test). Therefore, M2 differentially innervates the superficial layers, especially L1, of other cortical areas depending on the target area.

### SUBLAMINAR SEGREGATION OF iCC PROJECTIONS ORIGINATING FROM M2 L2/3 DEPENDS ON THE TARGET AREA

**FIGURE 8:**
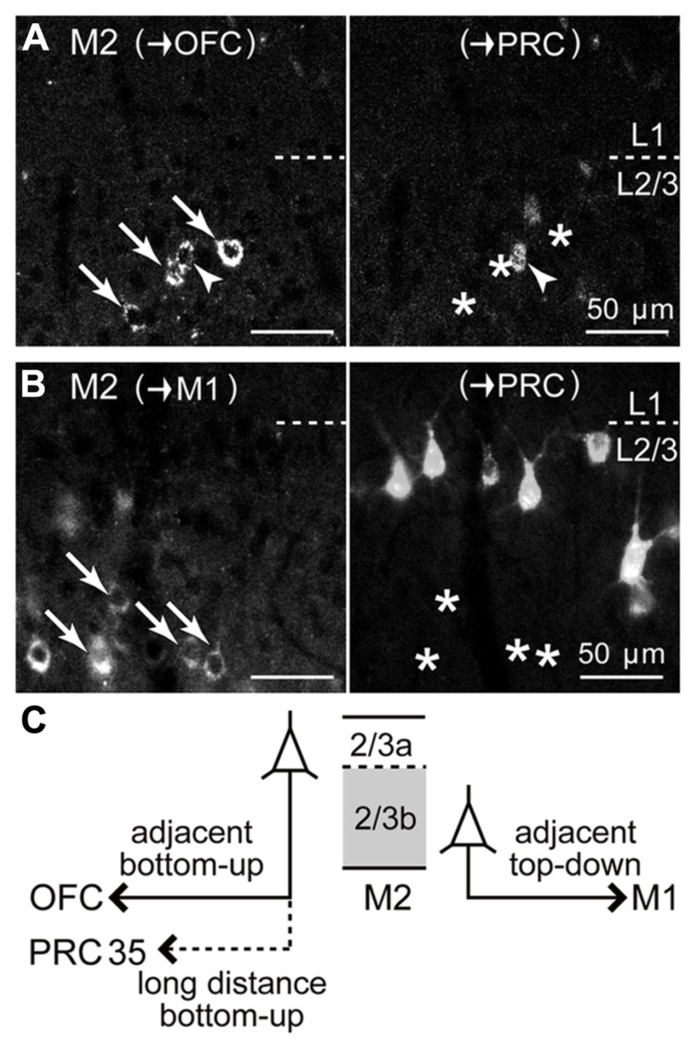
**Relation of iCC projections from M2 L2/3 to PRC with those to OFC or to M1.** **(A)** Upper L2/3 distributions of M2 iCC cells projecting to OFC and to PRC, and their partial double-labeling. Left, M2 somata retrogradely labeled from OFC (arrows and arrowhead; labeled by CTB555). Right, M2 somata retrogradely labeled from PRC (labeled by Fast Blue). Arrowhead, double-labeled cell; arrows, OFC-projecting cells not labeled by PRC injection (asterisks). Oblique section, 20 μm thickness. **(B)** Segregation of iCC projections from M2 L2/3 to M1 and to PRC. Left, M2 somata projecting to M1 in lower L2/3 (arrows; labeled by CTB555), not labeled from PRC (asterisks in right). Right, M2 somata projecting to PRC in upper L2/3 (labeled by Fast Blue). **(C)** Schematic summary of M2 L2/3 iCC projections. OFC-projecting cells are more prevalent in L2/3a, some of which also send axon collaterals to PRC. By contrast, M1-projecting cells are abundant in L2/3b.

Within L2/3 of M2, pyramidal cells projecting to OFC or PRC 35 were distributed more in L2/3a than in L2/3b, whereas those projecting to M1 were distributed more in L2/3b (**Figure 4**). L2/3 cells retrogradely labeled from PRC partially double-labeled those labeled from OFC (**Figure 8A**; two rats), but were almost entirely distinct from those labeled from M1, with a different sublaminar localization (**Figure 8B**; three rats). By contrast, L5a cells labeled from PRC partially overlapped with those labeled from M1 (data not shown; three rats). Some L2/3a cells projecting to PRC are also retrogradely labeled from the amygdala (Hirai et al., 2012). Therefore, M2 L2/3 sublayers are roughly correlated with different iCC systems: L2/3a projecting to OFC, PRC 35, and amygdala, and L2/3b projecting to M1 (**Figure 8C**).

## DISCUSSION

### COMPLEMENTARY LAMINAR DISTRIBUTIONS OF iCC CELLS RECIPROCALLY CONNECTING M2 AND NON-FRONTAL AREAS

We found that M2 and the distant non-frontal areas we examined were connected by pyramidal cells in either superficial or deep layers, depending on the direction of connectivity. In visual cortical areas, lower- to higher-order projections [called “forward (bottom-up) connections”] originate either from superficial layers or from both superficial and deep layers, and terminate in middle layers. By contrast, projections in the reverse direction [“backward (top-down) connections”] originate either from deep layers or from both superficial and deep layers and terminate outside middle layers, especially in L1 (Felleman and Van Essen, 1991; Rockland, 1997; Barone et al., 2000; Douglas and Martin, 2004; Shipp, 2007). Similar anatomical differences to the laminar patterns of iCC origins and their innervation sites between visual cortical areas have been found between sensory, motor, and association cortices (Felleman and Van Essen, 1991). Therefore, we assumed the directionality of reciprocal connections between M2 and its target areas by analogy with the directionality demonstrated between visual areas.

**FIGURE 9:**
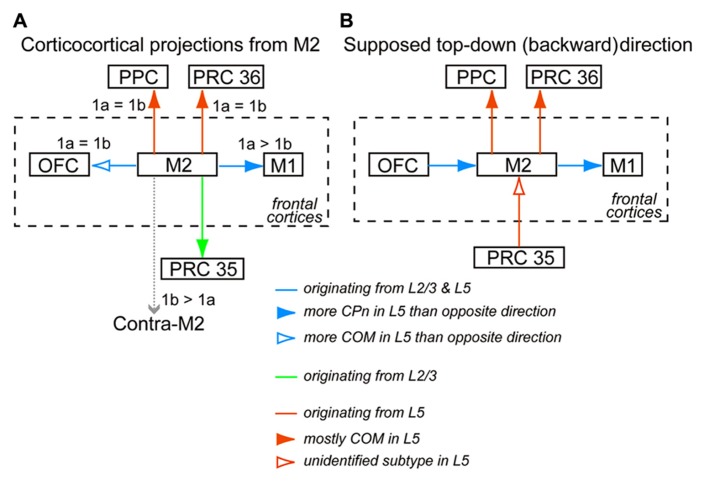
**Interareal direction-dependent laminar distributions of iCC cells and involvement of L5 pyramidal cell subtypes. (A)** iCC projections from M2 and their L1 innervation in the target area. iCC connections to the adjacent areas are mediated by L2/3 cells as well as L5 cells of both CPn and COM subtypes, but their relative involvement depends on each connection. More fibers from M2 terminate in L1a than in L1b of M1 (1a > 1b), but they terminate in L1a and L1b of OFC at similar levels (1a = 1b). iCC connections to the distant areas are mediated by either L2/3 cells or L5 cells of mostly COM subtypes. Fibers from M2 terminate at comparable levels in L1a and L1b of PRC 36 and PPC. By contrast, more fibers from M2 terminate in L1b than in L1a of contralateral M2 (1b > 1a). **(B)** Supposed top-down (backward) connections, assuming more involvement of L5 cells than L2/3 cells in that direction between M2 and the distant non-frontal areas, and more involvement of L5 CPn cells than COM cells in that direction between M2 and the adjacent areas.

Between M2 and a distant area, including PRC 35, PRC 36, and PPC, the laminar patterns of iCC cells were highly complementary (**Figures 4D,E**). Between M2 and PPC, the direction from PPC to M2 was considered bottom-up, while that from M2 to PPC was considered top-down (**Figure 9**). The frontal cortex receives visual inputs through the parietal cortex, not directly from the visual cortex, to control visuomotor and attentional performance (Wise et al., 1997; Reep and Corwin, 2009; Corbetta and Shulman, 2011). According to the anatomical connectivity, supposed bottom-up signals from PPC relaying visual information would arrive at M2. By contrast, the anatomical connectivity between M2 and PRC 36 was the reverse of that between M2 and PRC 35. M2-to-PRC 35 and PRC 36-to-M2 projections were considered bottom-up connections (**Figure 9**). Signals from the frontal cortex to the hippocampal formation may initiate active retrieval of declarative memories (Miyashita, 2004). According to the anatomical connectivity, supposed retrieval signals would arrive at PRC 35, with stronger connections with the entorhinal cortex than PRC 36 (Burwell and Amaral, 1998; Agster and Burwell, 2009). Meanwhile, the retrieved memory would be transmitted to the frontal cortex from PRC 36, which is more strongly connected to sensory and temporal cortical areas than PRC 35 (Burwell and Amaral, 1998; Agster and Burwell, 2009).

### DIFFERENTIATION OF L5 PYRAMIDAL CELLS ACCORDING TO DIVERSE TELENCEPHALIC AND SUBCEREBRAL PROJECTIONS

**FIGURE 10:**
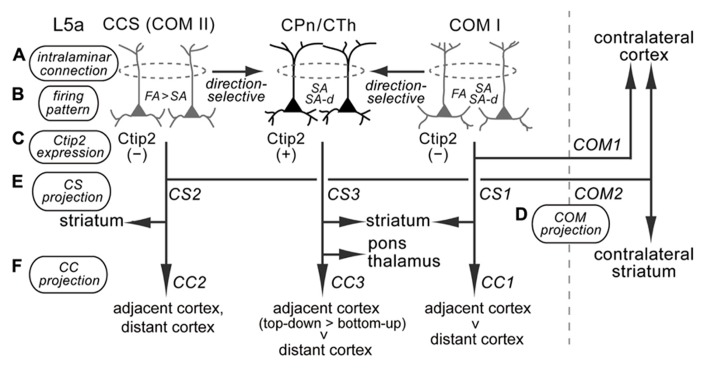
**Diversity of supposed relationships among iCC projections, with multiple corticostriatal and COM cell subtypes in M2 L5a.** M2 L5a contains CPn/CTh, COM type I, and COM type II/CCS cells that differ in their morphological, physiological, and connectional characteristics, and all of these cells send axon collaterals to the ipsilateral striatum (Morishima and Kawaguchi, 2006; Morishima et al., 2011; Otsuka and Kawaguchi, 2011; Hirai et al., 2012). **(A)** Intralaminar connection pattern: CPn cells innervate other CPn cells, and COM cells form synaptic connections particularly with other COM cells sharing the same firing pattern (Morishima et al., 2011; Otsuka and Kawaguchi, 2011). COM subtypes innervate CPn cells, but CPn cells rarely innervate COM subtypes (Morishima and Kawaguchi, 2006; Kiritani et al., 2012). **(B)** Firing pattern: CPn cells and COM subtypes contain different proportions of FA, SA, and SA-d firing types (Otsuka and Kawaguchi, 2008, 2011; Hirai et al., 2012; the present study). **(C)** Ctip2 expression: CPn/CTh cells specifically express Ctip2, but COM cells do not (Arlotta et al., 2005; Ueta et al., 2013). **(D,E)** Corticostriatal (CS) and COM projection patterns: COM type I cells project to the contralateral cortex (COM1) and ipsilateral striatum (CS1); CCS (COM type II) cells project to the contralateral striatum in addition to the contralateral cortex (COM2) and ipsilateral striatum (CS2); and CPn/CTh cells project to the ipsilateral striatum (CS3), but not to the contralateral hemisphere (Lévesque et al., 1996; Lévesque and Parent, 1998; Otsuka and Kawaguchi, 2011; Hirai et al., 2012; Shepherd, 2013). **(F)** iCC projection pattern: COM type I cells preferentially innervate the adjacent cortex compared to the distant cortex (CC1); COM type II cells project to various adjacent and distant cortices; and CPn/CTh cells preferentially innervate the adjacent cortex (especially in the top-down direction) rather than the distant cortex (the present study).

L5a pyramidal cells participate in diverse iCC projections from M2 to multiple cortical areas. Based on the projection patterns to subcortical structures and the contralateral cortex, as well as firing characteristics and Ctip2 molecular expression, L5a pyramidal cells could be divided into at least three subtypes (**Figure 10**). Importantly, these subtypes are also differentially involved in iCC connections.

In addition to their innervation of the pontine nuclei, CPn cells also project to other subcortical targets according to their depth location within L5 of M2: CTh cells without spinal cord innervation in L5a; CTc cells without spinal cord innervation in lower L5a; CSp cells in L5b, some of which innervate the thalamus; and CTc cells with spinal cord innervation in upper L5b. The frontal cortex also sends axon collaterals to the subthalamus (Nambu et al., 2002; Kita and Kita, 2012). The subthalamus-projecting cells are a subtype of L5 CPn cells that also innervate the thalamus and superior colliculus (Kita and Kita, 2012) and are distributed in the middle of L5, consistent with the laminar distribution of CTc cells (**Figure 2B**). This anatomical distribution suggests that CPn cells at a given depth share the same extracortical targets and that CPn cells may differentiate strongly depending on cortical depth. COM cells may be more diverse at a given depth, as the same sublayer contains at least two subtypes of COM cells that differ in physiological, morphological, and projection characteristics (**Figure 10**; Otsuka and Kawaguchi, 2011).

**FIGURE 11:**
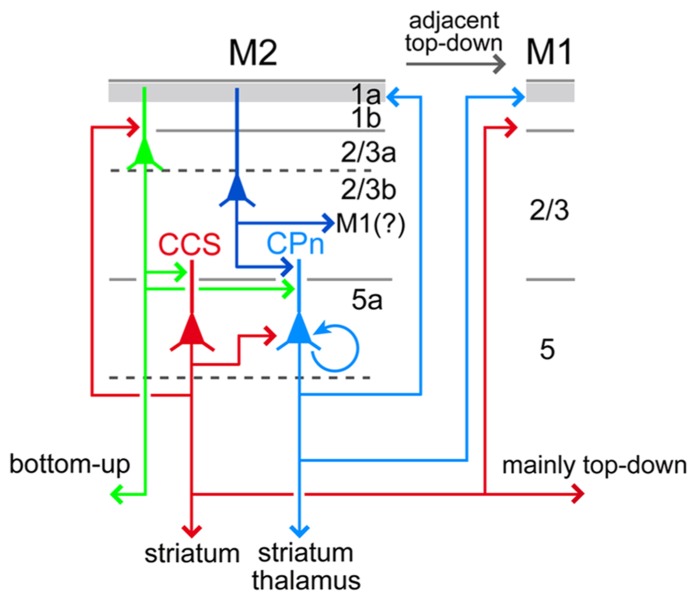
**Relationships between local excitation loops involving L5a CCS and CPn cells with iCC projections.** L5a CCS cells innervate CPn cells unidirectionally in L5 and may form excitatory loops with L2/3a cells including OFC-projecting (bottom-up projection to an adjacent area) and PRC 35-projecting cells (bottom-up projection to a distant area). L5a CCS cells send axon collaterals to various cortical areas and both sides of striatum, but not to PRC 35. By contrast, CPn cells develop more reciprocal connections with each other than CCS cells, innervate L1a preferentially over L1b/L2/3a in their own area as well as in ipsilateral M1, and receive convergent inputs from L2/3 cells of various depths. L5a CPn cells send outputs to the ipsilateral thalamus as well as striatum. Apical dendrites of L5a CCS and CPn cells extending to L1a are omitted (see **Figure 10**).

Both CPn and COM cells in L5 participate in iCC projections. In M2, unlike M1, L5 iCC projections originate mainly from L5a (**Figure 4D**; Ueta et al., 2013). L5a CPn cells innervate the adjacent frontal areas, but weakly innervate the distant areas. The involvement of L5a COM cells in iCC connections differs between their subtypes: the SA subtype may participate mainly in adjacent areas, but the FA subtype, including CCS cells, participates in both intrafrontal and distant projections (**Figures 10** and **11**; Otsuka and Kawaguchi, 2008, 2011; Hirai et al., 2012; Ueta et al., 2013). These findings suggest that L5a CPn cells and COM cells with similar firing characteristics to CPn cells share common iCC innervation territory distinct from that of CCS cells, which have a wider area of innervation (**Figures 10** and **11**). For a deeper understanding of the functional interactions of iCC communication with subcortical and COM projections, it would be important to examine their relationships more quantitatively by introducing selective molecular markers for individual neuron subtypes (Molnár and Cheung, 2006; Molyneaux et al., 2007; Fame et al., 2011).

### DIRECTION-DEPENDENT INVOLVEMENT OF CPN CELLS IN iCC CONNECTIONS BETWEEN FRONTAL AREAS

Among adjacent frontal areas, M2 is connected not only with M1, but also with OFC (Conte et al., 2008; Reep and Corwin, 2009; Hoover and Vertes, 2011). Since these frontal areas were bidirectionally connected by pyramidal cells in both superficial and deep layers (**Figures 4D,E**), the directional connectivity could not be determined solely based on laminar patterns of iCC origins.

L5 pyramidal cells connecting ipsilateral frontal areas were found in both L5a and L5b, and contained both Ctip2-positive CPn cells and Ctip2-negative COM cells, with their selective sublaminar distribution and Ctip2 expression patterns dependent on the connection direction (**Figures 4D,E** and **5**). M2 innervates L1a of M1 more preferentially than M1 innervates M2 (**Figure 7B**), and the L1a innervation is conveyed by Ctip2-positive cells in M2 L5a, including CTh cells (**Figure 11**; Ueta et al., 2013). Between visual cortical areas, the backward projections innervate upper L1 to a greater degree than the forward projections (Coogan and Burkhalter, 1993; Dong et al., 2004). To understand the CC connections in a unified framework, we assumed that, in reciprocal connectivity between frontal areas, the direction with more involvement of Ctip2-positive L5 cells, from OFC to M2 and from M2 to M1, was defined as top-down (**Figure 9**). Therefore, frontal areas might be directionally connected via L5a CPn cells with more L1 innervation in a rostral-to-caudal, top-down direction. L5a CPn cells are involved in projections to the adjacent ipsilateral cortical areas as well as the ventral thalamic nuclei, whereas L5b CPn cells send specific outputs from individual areas to the thalamus, brainstem, and spinal cord (**Figure 2**).

The framework for iCC connectivity of the frontal cortex proposed here is similar to the idea that descending projections from the motor cortex are more like backward connections in the visual cortex than the corresponding forward connections (Shipp, 2005). It is proposed that descending connections of sensory cortices convey predictions of sensory inputs, and by the same token those of frontal cortices send proprioceptive predictions, rather than motor commands (Adams et al., 2013).

### DIVERSE LAMINAR INNERVATION PATTERNS BY L5 PYRAMIDAL CELL SUBTYPES IN THE TARGET CORTEX

COM cells in M2 preferentially innervate L1b and L2/3 compared to L1a in contralateral M2 (**Figure 7E**), whereas L5a CPn cells in M2 prefer to send axons to L1a within M2 and in M1 (Hirai et al., 2012; Ueta et al., 2013). Therefore, when both L5a COM and CPn cells in M2 project to another cortical area, two types of M2 L5 activity may be transferred independently into the local circuit of the target cortex. L5a CPn cells in the source area send axon collaterals to L1a in the iCC target area, where they interact with axon collaterals in L1a arising from L5 CPn cells in the target area (Thomson and Bannister, 2003), as well as with thalamocortical innervations relaying basal ganglia outputs that heavily terminate in L1a (Kuramoto et al., 2009, 2011; Rubio-Garrido et al., 2009; Kaneko, 2013). Similarly, axon collaterals of L5a COM cells in the source area would interact with those in the target area at L1b and L2/3. These observations suggest that the activities of L5a CPn and COM cells are separately processed both in their own and iCC target areas.

### SUBLAMINAR DISSOCIATION OF L2/3 BOTTOM-UP AND TOP-DOWN iCC PROJECTIONS IN M2

In L2/3 of M2, iCC cells projecting to both OFC and PRC 35 originate mainly from L2/3a, whereas those projecting to M1 originate from L2/3b (**Figure 8**). The former type of projection also sends axon collaterals to the amygdala (Hirai et al., 2012). Therefore, in superficial layers, iCC projections separately originate from the upper and lower L2/3 sublayers according to their targets. Considering the connectivity between M2 and its target cortical areas, the bottom-up projection may originate from L2/3a, whereas the top-down projection may originate from L2/3b.

Both SA and SA-d firing types of L5 pyramidal cells receive strong feedforward excitation from L2/3 pyramidal cells (Thomson and Bannister, 2003; Yu et al., 2008; Petreanu et al., 2009) irrespective of their L2/3 depth (Otsuka and Kawaguchi, 2008), indicating convergent inputs to CPn cells from L2/3 cells. By contrast, FA-type pyramidal cells in upper L5 (probably corresponding to L5a) receive excitation from upper L2/3 (L2/3a) pyramidal cells, whereas those in middle L5 (upper L5b) receive excitatory inputs from middle L2/3 (probably upper L2/3b), indicating that interlaminar connections are topographically organized depending on L5 pyramidal cell subtypes (Otsuka and Kawaguchi, 2008; Anderson et al., 2010; Hirai et al., 2012). Interestingly, this finding suggests that M2 L5 CCS cells can be differentiated into L5a cells receiving excitatory input from L2/3a cells that may carry bottom-up signals, and upper L5b cells receiving excitatory input from L2/3b cells that may carry top-down signals.

L5a CCS cells reciprocally connect with L2/3a pyramidal cells, and their axodendritic contacts are located in L1b and L2/3 (**Figure 11**; Hirai et al., 2012). Therefore, the excitation loop between L2/3a pyramidal cells and L5a CCS cells is important for the integration of the long-distance bottom-up and top-down iCC connections in higher-order frontal motor areas (**Figure 11**). Following the firing of CCS cells, L5a CPn cells would be activated by unidirectional CCS connections and, if sufficiently excited, maintain persistent firing via facilitating reciprocal excitation among CPn cells (**Figure 11**; Morishima and Kawaguchi, 2006; Morishima et al., 2011; Morita et al., 2012). Furthermore, if a sufficient number of CPn cells fired persistently, L2/3 pyramidal cells could begin to fire tonically in response to depolarization evoked by ascending axon collaterals of L5 CPn cells to L1a, targeting distal tufts of L2/3 pyramidal cells. Increased firing of L2/3 pyramidal cells would subsequently facilitate their convergent outputs to L5 CPn cells (**Figure 11**).

We have classified frontal cortical pyramidal cells into several major classes based on their anatomical and physiological properties. It is likely that future studies examining selective expression of molecular markers in these populations may reveal even finer subtype specialization. However, our current work demonstrates a fundamental structural relationship between local circuit elements and long-distance top-down and bottom-up connectivity within the frontal cortex that may reflect a fundamental organizing principle of the cerebral cortex as a whole.

## CONCLUSION

We identified individual iCC connections between M2 and other areas as being “top-down” or “bottom-up” by comparing the laminar distribution of iCC cells and their efferent innervations, COM/subcortical projections of these neurons, and their firing patterns. Based on our results, we proposed a provisional unified framework of interareal hierarchy within the frontal cortex and between the fontal and non-frontal areas. Furthermore, we discussed the functional interaction of the interareal hierarchy with the intraareal local cortical microcircuit.

## Conflict of Interest Statement

The authors declare that the research was conducted in the absence of any commercial or financial relationships that could be construed as a potential conflict of interest.

## Footnotes

1 http://www.anim.med.kyoto-u.ac.jp:80/nbr/default.aspx

2 http://www.neuromatic.thinkrandom.com
